# Accessible Silver-Iron Oxide Nanoparticles as a Nanomaterial for Supported Liquid Membranes

**DOI:** 10.3390/nano11051204

**Published:** 2021-05-01

**Authors:** Ioana Alina Dimulescu (Nica), Aurelia Cristina Nechifor, Cristina Bǎrdacǎ (Urducea), Ovidiu Oprea, Dumitru Paşcu, Eugenia Eftimie Totu, Paul Constantin Albu, Gheorghe Nechifor, Simona Gabriela Bungău

**Affiliations:** 1 Analytical Chemistry and Environmental Engineering Department, University Politehnica of Bucharest, 1-7 Polizu Street, 011061 Bucharest, Romania; oanaalinadimulescu@yahoo.com (I.A.D.); cristinabardaca@yahoo.com (C.B.); dd.pascu@yahoo.com (D.P.); gheorghe.nechifor@upb.ro (G.N.); 2 Department of Inorganic Chemistry, Physical Chemistry and Electrochemistry, University Politehnica of Bucharest, 1-7 Polizu Street, 011061 Bucharest, Romania; ovidiu.oprea@upb.ro; 3 Radioisotopes & Radiation Metrology Department (DRMR), Horia Hulubei National Institute for R&D in Physics and Nuclear Engineering (IFIN-HH), 30 Reactorului Street, 023465 Magurele, Romania; paulalbu@gmail.com; 4 Faculty of Medicine and Pharmacy, University of Oradea, Universităţii Street No.1, 410087 Oradea, Romania; sbungau@uoradea.ro

**Keywords:** silver recovery, Ag–Fe oxide nanoparticles, nanoparticles electrochemical synthesis, magnetic nanocarriers, liquid membranes

## Abstract

The present study introduces the process performances of nitrophenols pertraction using new liquid supported membranes under the action of a magnetic field. The membrane system is based on the dispersion of silver–iron oxide nanoparticles in n-alcohols supported on hollow microporous polypropylene fibers. The iron oxide–silver nanoparticles are obtained directly through cyclic voltammetry electrolysis run in the presence of soluble silver complexes ([AgCl_2_]^−^; [Ag(S_2_O_3_)_2_]^3−^; [Ag(NH_3_)_2_]^+^) and using pure iron electrodes. The nanostructured particles are characterized morphologically and structurally by scanning electron microscopy (SEM and HFSEM), EDAX, XRD, and thermal analysis (TG, DSC). The performances of the nitrophenols permeation process are investigated in a variable magnetic field. These studies show that the flux and extraction efficiency have the highest values for the membrane system embedding iron oxide–silver nanoparticles obtained electrochemically in the presence of [Ag(NH_3_)_2_]^+^ electrolyte. It is demonstrated that the total flow of nitrophenols through the new membrane system depends on diffusion, convection, and silver-assisted transport.

## 1. Introduction

Bart Van der Bruggen underlined the impact of the nanomaterials’ development on the domain of membranes and membrane processes at the European Membrane Society Summer School, Bucharest 2010 when he stated that “the development of membranes research in the next decade will be marked by the influence of various nanomaterials on obtaining membranes and membrane processes, but especially on their applications” [1]. Nowadays, after more than a decade, there is an excellent perspective on the benefits of various nano species, often with chemical changes, over the specific domain of membranes [2].

Many nano species have been used in the preparation of membranes or processes involving membranes and their various applications, as follows: nano species derived from carbon (carbon nanotubes, fullerenes, graphene) [2,3]; metallic nanoparticles (silver, gold, copper) [4,5]; oxide nanoparticles (silica, titanium dioxide, iron oxides, copper oxide, zinc oxide, aluminum oxide) [6,7,8,9]; polymeric nanoparticles (ion exchangers, reactive and complexed polymers) [10,11]; proteins, and enzymes [12]. Most of the time, the effect of nano species used in membranes and membrane processes was defined, and their preparation or cost did not condition any application. An important number of researchers developed different methods for obtaining the same nanomaterials, but with as many diverse and controllable characteristics as possible [13,14].

Notably, the iron oxide nanoparticles with magnetic properties (maghemite or magnetite) generated a particular interest for studies on advanced membranes based on nanomaterials [13,14,15,16,17].

For this purpose, a multitude of methods for obtaining the magnetic nanoparticles of magnetite are known today, including the co-precipitation method [18], sol–gel process [19], thermal decomposition [20], solvothermal procedure [21], hydrolysis and thermolysis of precursors [22], sonochemical method [23], electrodeposition [24], hydrothermal reactions [25], flow injection [26], microemulsion reactions [27], flame spray pyrolysis [28], vapor deposition [29], and microwave [30].

Among these possible synthesis ways, the electrochemical procedure has the advantage of the necessary equipment simplicity and the versatility of its experimental parameters: potential, current density, pH, organic or inorganic additives [31]. The electrochemical method allows obtaining various magnetic iron oxides in different electrolytes, while the productivity could be controlled [32,33,34,35].

In the meantime, the interest in silver usage as an antimicrobial agent has led to many membranes that incorporate silver nanoparticles to prevent microbial growth on surfaces [36]. Consequently, obtaining silver nanoparticles through chemical or physical procedures is still the core of considerable research [37]. From the point of view of chemical methods, various reducing agents as sodium citrate, ascorbic acid, thiosulfate, sodium borohydride (NaBH_4_), elemental hydrogen, polyols, Tollens reagent, N, N-dimethylformamide (DMF), and copolymers with poly(ethylene glycol) block, hydrazine, and ammonium formate are used to reduce silver ions (Ag^+^) in aqueous or non-aqueous solutions [38,39].

The green synthesis of nanoparticles has also received considerable attention due to the growing need to develop clean technologies to synthesize materials [40]. Thus, an enormous effort was directed towards the green synthesis of inorganic materials, especially metal nanoparticles using microorganisms and plant extracts [41].

The use of silver and/or iron nanoparticles in membranes and membrane processes became traditional and has not stopped developing [42,43,44,45].

Liquid membranes are based on a double liquid–liquid extraction system in equilibrium, in which the role of the membrane is played by a solvent immiscible with water [46,47]. This type of membrane has several advantages like minimization of solvent circulated in the extraction process; possibility to separate solutions of various sizes; ease of operating; facile determination of the transport characteristics for the solutions subjected to separation; optimization of the membrane processes by increasing the selectivity, the separation and concentration yields through the membrane’s carriers.

The separation, purification, or concentration of various organic compounds through the liquid membranes faces a continuous development assured by the qualities of this type of membranes: high and directed selectivity, advantageous possibilities of scaling, permanent improvement of process engineering [46,47,48,49].

The significant problems that need to be permanently addressed for applying the liquid membranes in various separation, purification, or concentration technologies are the reduction of the organic phase (membranes) volume, the attainment of a mass transfer surface as large as possible, and high stability in various aqueous environments [50,51,52,53].

Nanoparticle dispersions, including silver and iron oxides, can increase the separation performances of the systems with supported liquid membranes. Such nanoparticles could be obtained through electrochemical methods (clean procedure and easy-to-conduct parameters) and silver chloride, a recovered raw material, for synthesis. The present work aimed to obtain liquid dispersion membranes based on affordable magnetic silver–iron oxide nanoparticles electrochemically synthesized. The prepared liquid membranes containing *n-*octanol and *n-*decanol were supported on microporous polypropylene fibers. Subsequently, the membranes were used to transport *o-* and *m-*nitrophenols in the presence of an oscillating magnetic field.

## 2. Experimental

### 2.1. Materials

The materials used in the presented work were of analytical grade. They were purchased from Merck (Merck KGaA, Darmstadt, Germany): ammonia, sodium thiosulfate, sodium hydroxide, hydrochloric acid, and from Sigma-Aldrich (Merck KGaA, Darmstadt, Germany): *o-* and *m-*nitrophenol, *n-*octyl alcohol (molar mass: 130.23 g/mol, density: 830 kg/m³, solubility in water: 0.300 g/L), *n*-decyl alcohol (molar mass: 158.28 g/mol, density: 830 kg/m³, solubility in water: 0.037 mg/L). The purified water characterized by 18.2 μS/cm conductivity was obtained with a RO Millipore system (Milli-Q^®^ Direct 8 RO Water Purification System, Merck, Darmstadt, Germany). The tubular dialysis membranes were from Visking (Medicell Membranes Ltd., London, UK).

**Figure 1:**
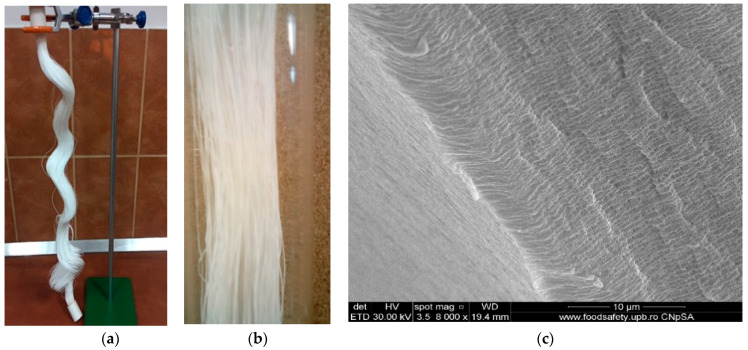
Polypropylene hollow fiber: (**a**) hollow fiber bundle; (**b**) detail; (**c**) individual hollow fiber cross-section (SEM image).

The hollow polypropylene fibers used as membranes’ support were provided by GOST Ltd., Perugia, Italy (Table 1, see Figure 1) [54,55].

The packing procedure of polypropylene hollow fibers in a bundle (Figure 1a) allows the achievement of the desired separation surface, in our case 1.0 m^2^, and the easy realization of the scaling by inserting several fascicles in flexible permeation modules [56]. The details (Figure 1b) presented to show the pore dimensions evenly distributed in the membrane fiber section (Figure 1c). The highlighted characteristics allow the impregnation of polypropylene hollow fibers with solutions or dispersions of nanoparticles [55,56,57].

### 2.2. Procedures

#### 2.2.1. Preparation of Silver–Iron Oxide Nanoparticles

The silver chloride residues coming from the didactic and research laboratories of the Faculty of Applied Chemistry and Materials Science from the Polytechnic University of Bucharest were filtered and then subjected to dialysis, in a continuous regime, up to neutral pH.

The recovered silver chloride was used in three parts of 50 g each as follows:

– One part was solubilized with 10% hydrochloric acid solution (gravimetrically);
– The second part was solubilized with sodium thiosulfate 10% solution (gravimetrically);
– The third part was solubilized with ammonia 10% solution (gravimetrically).

A 250 mL volume of each solution was introduced, in turn, into an electrolysis cell of the PARSTAT 2273 potentiostat equipment provided with three electrodes: pure iron anode and cathode, and a platinum wire as a reference. Cyclic voltammetry was performed for a potential sweep between −0.5 and +1.23 V at a scan rate of 50 mV/s. The experimental procedure took place at room temperature.

After six hours of work at the cell base, black nanoparticles were collected. Afterward, they were magnetically transferred into a dialyzer with a regenerated cellulose membrane. Continuous dialysis was performed until it was obtained a chloride-free sample. The chloride anion concentration was followed up with a combined selective chloride electrode (HI 4107, Hanna Instruments Ltd., Leighton Buzzard, UK) and a multiparameter system (HI5522, Hanna Instruments Ltd., Leighton Buzzard, UK).

The nanocomposite samples containing iron oxide and silver were characterized by scanning electron microscopy (SEM and HFSEM), EDAX, XRD, UV-Vis, atomic absorption spectroscopy, and thermal analysis (TG, DSC).

#### 2.2.2. Obtaining Liquid Membranes on Polypropylene Support

A volume of 482 mL (about 400 g), *n*-alcohol (*n*-octanol or *n*-decanol), and 20 g of silver–iron oxide nanoparticles were placed in an 800 mL glass tank. The glass tank was placed in an ultrasonic bath (Elmasonic S, Elma Schmidbauer GmbH, Singen, Germany) for two hours, observing the complete dispersion. Finally, a dark brown dispersed liquid system was formed.

The bundle of microporous polypropylene fibers was immersed in the obtained dispersion without inserting the sealing ends (made of polyvinyl chloride). After 24 h, the bundle of polypropylene fibers impregnated with nanoparticles containing iron oxide and silver in the considered n-alcohol is recovered and washed for 10 minutes with deionized water—see Figure S1 in Supplementary Materials [56,57].

#### 2.2.3. Pertraction of *o*- and *m*-Nitrophenols with Impregnated Liquid Membranes

**Figure 2:**
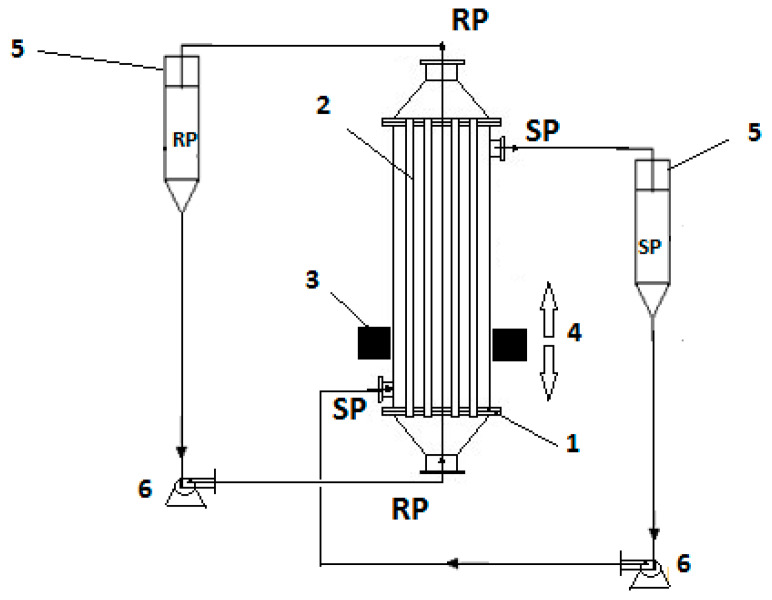
Schematic presentation of the pertraction module and its principal components: 1–pertraction module, 2—hollow-fiber membrane, 3—annular ferrite, 4—oscillatory system, 5—reservoirs: SP-source phase, RP-receiving phase, 6-pumps.

The impregnated membrane bundle was mounted in a permeation module similar to that extensively described in our previous works [54,55,57] (Figure 2), excepting the annular ferrite. Thus, inside an annular ferrite (Figure S2d in Supplementary Materials) was introduced a glass resistant tube (800 mm × 60 mm), after which the inlet and outlet of the fluids were fixed on the side (at 50 mm from the ends). A bundle of impregnated fibers was inserted in this casing and subsequently immobilized with acrylic polymer, thus obtaining a permeator similar to a tubular heat exchanger. This module was placed in the separation equipment (Figure 2).

The transport of *o*- and *p*-nitrophenols was performed using the above-described permeation installation that allowed the circulation of the source phase outside the membrane bundle and the receiving phase inside the hollow fibers (Figure 2). The used tanks had 10 L for the supply solution and 2 L for the receiving solution. The annular ferrite placed outside the permeation module could translate, up and down, around the permeator with a frequency of 10–60 cycles per minute.

The central element of the installation is the fiber bundle (Figure S2a). When the fibers are impregnated, they are attracted by the magnetic field created by the working ferrite (Figure S2b) or other magnetic material (Figure S2c). The geometric characteristics of the working ferrite were provided by the manufacturing company (Figure S2d).

The source phase (SP) made of nitrophenol synthetic solution, with a concentration of 2 g/L and pH = 1 [58,59], was introduced in the installation where the hollow-fiber membrane assured an effective mass transfer surface of 1.0 m^2^. The receiving phase (RP) was formed by a 0.1 mol/L sodium hydroxide solution [60]. Three samples of 1 mL from the SP aqueous nitrophenol synthesized solutions were periodically spectrophotometrically analyzed (CamSpec spectrophotometer) [59,60].

The fluxes from the source phase [61,62] were determined against the measured permeate mass within a determined time range, applying the following equation:

(1) $$J=\frac{M}{S\cdot t}\left(g/m^{2}h\right)$$

where: *M* = permeate mass (g); *S* = the effective surface of the membrane (m^2^); *t* = the time necessary to collect the permeate volume (h).

The extraction efficiencies (*EE*%) of analytes calculated using the solutions’ concentration or absorbance [63] were:

(2) $$EE\left(\%\right)=\frac{\left(c_{0}-c_{f}\right)}{c_{0}}\cdot 100$$

where: *c*_f_—the final concentration of the solute (nitrophenol); *c*_o_—initial concentration of solute (nitrophenol):

(3) $$EE\left(\%\right)=\frac{\left(A_{0}-A_{s}\right)\;}{A_{0}}\cdot 100$$

where: *A*_0_—initial sample solution absorbance; *A*_s_—current sample absorbance.

### 2.3. Equipment

The microscopy studies, SEM and HFSEM, were performed on a Hitachi S4500 system (Hitachi High-Technologies Europe GmbH, Krefeld, Germany). Thermal characterizations were performed on a Netzsch thermal analyzer (NETZSCH-Gerätebau GmbH, Selb, Germany). The thermal analysis was run in a nitrogen atmosphere at a 10 °C/min heating rate, from the room temperature (RT = 25 °C) up to 900 °C.

The UV-vis analyses of the aqueous nitrophenol solutions were done on a spectrometer CamSpec M550 (Spectronic CamSpec Ltd., Leeds, UK).

The electrochemical processes were followed up with a PARSTAT 2273 Potentiostat (Princeton Applied Research, AMETEK Inc., Oak Ridge, TN, USA). We used a setup with a glass cell with three electrodes.

XRD analyses were recorded using PANalytical X’Pert Pro MPD equipment (PANalytical B.V., Almelo, The Netherlands) with a CuKα radiation source, and 2θ measurement range from 10° to 90°.

The nanoparticle magnetization diagrams were determined with Quantum Design MPMS 3 Magnetometer (Quantum Design Europe, Darmstadt, Germany) based on superconducting quantum interference device detection (SQUID). The DC operation mode applied allowed to run SQUID-vibrating-sample magnetometer (VSM) measurements.

The UV-vis studies on the nanoparticles samples were performed on dual-beam UV equipment—Varian Cary 50 (Agilent Technologies Inc., Wood Dale, IL, USA) at a resolution of 1 nm, spectral bandwidth 1.5 nm, and 300 nm/s scan rate. The samples’ UV-Vis spectra were recorded for a wavelength from 200 to 800 nm, at room temperature, using 10 mm quartz cells.

To assess and validate the content in metal ions, an atomic absorption spectrometer AAnalyst 400 AA spectrometer (PerkinElmer Inc., Shelton, CT, USA) with WinLab32—AA software (PerkinElmer), with a single-element hollow-cathode lamp was used. The operating current was set up at 2 mA, wavelength 248.3 nm, and 0.2 nm spectral bandwidth for determining the iron content. For silver, the experimental parameters were 328.1 nm wavelength and 0.7 nm spectral bandwidth at an operating current of 5 mA.

## 3. Results

**Figure 3:**
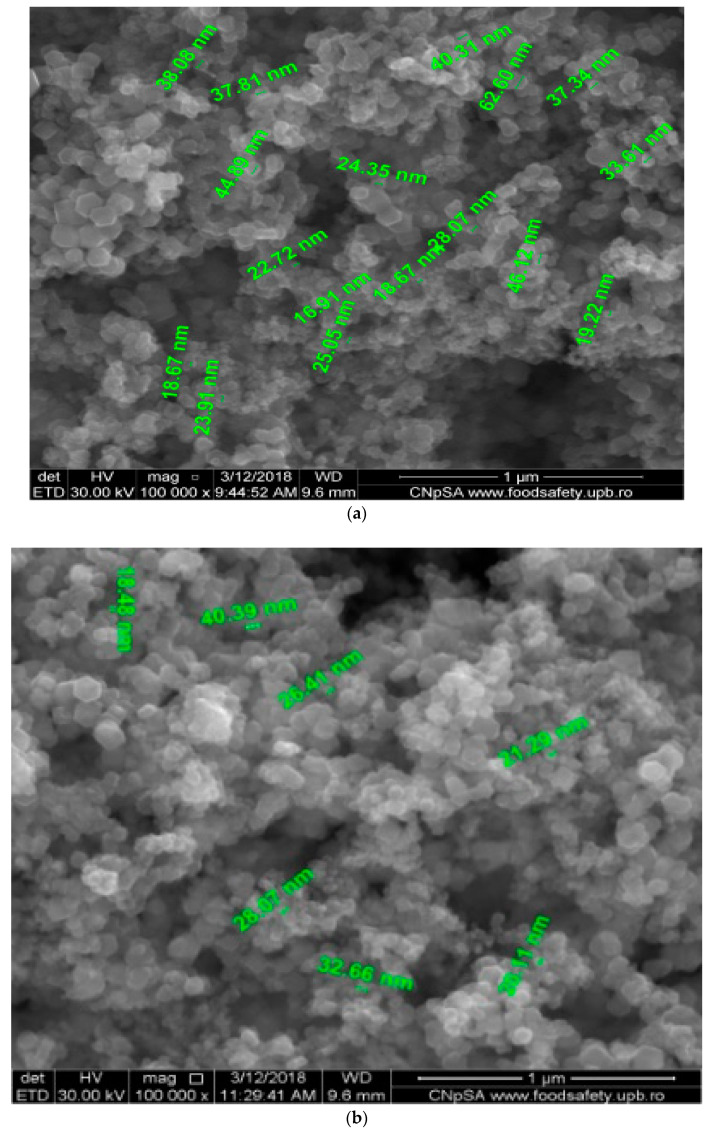

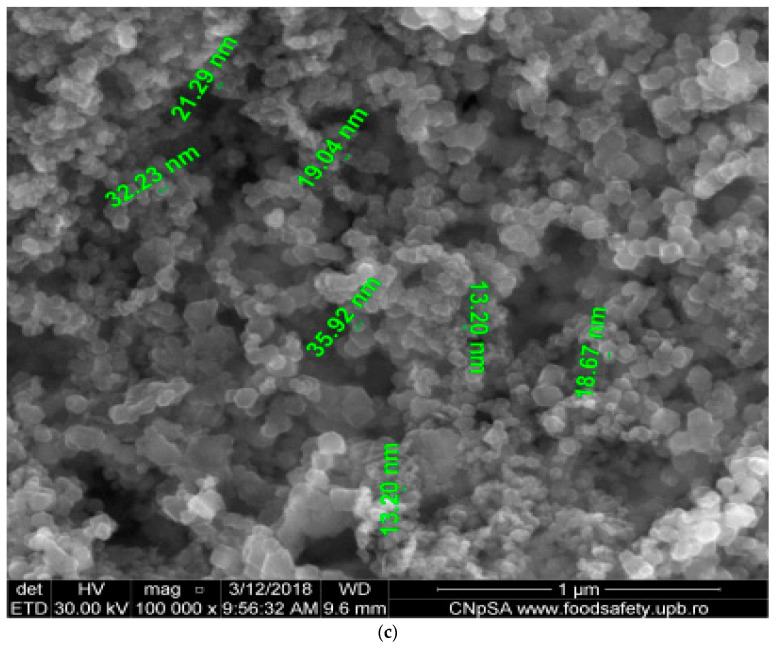
SEM images. (**a**) NP_1_ morphologies (**b**) NP_2_ morphologies; (**c**) NP_3_ morphologies.

Figure 3 presents the scanning electron microscopy images for the composite nanoparticles obtained using the electrolytic systems based on:

[AgCl_2_]^−^ shorthand notation NP_1_;

[Ag(S_2_O_3_)_2_]^3−^ shorthand notation NP_2_;

[Ag (NH_3_)_2_]^+^ shorthand notation NP_3_.

The images from Figure 3a–c present the particles’ morphologies and their dimensions. The EDAX diagrams depicted in Figure S3a–c from Supplementary File reflect the surface composition of the nanoparticles.

XRD patterns for the obtained nanoparticles are presented in Figure S4 (Supplementary File). Comparing the experimental data to reference fingerprints, it was determined which phases are present in the analyzed samples.

**Figure 4:**
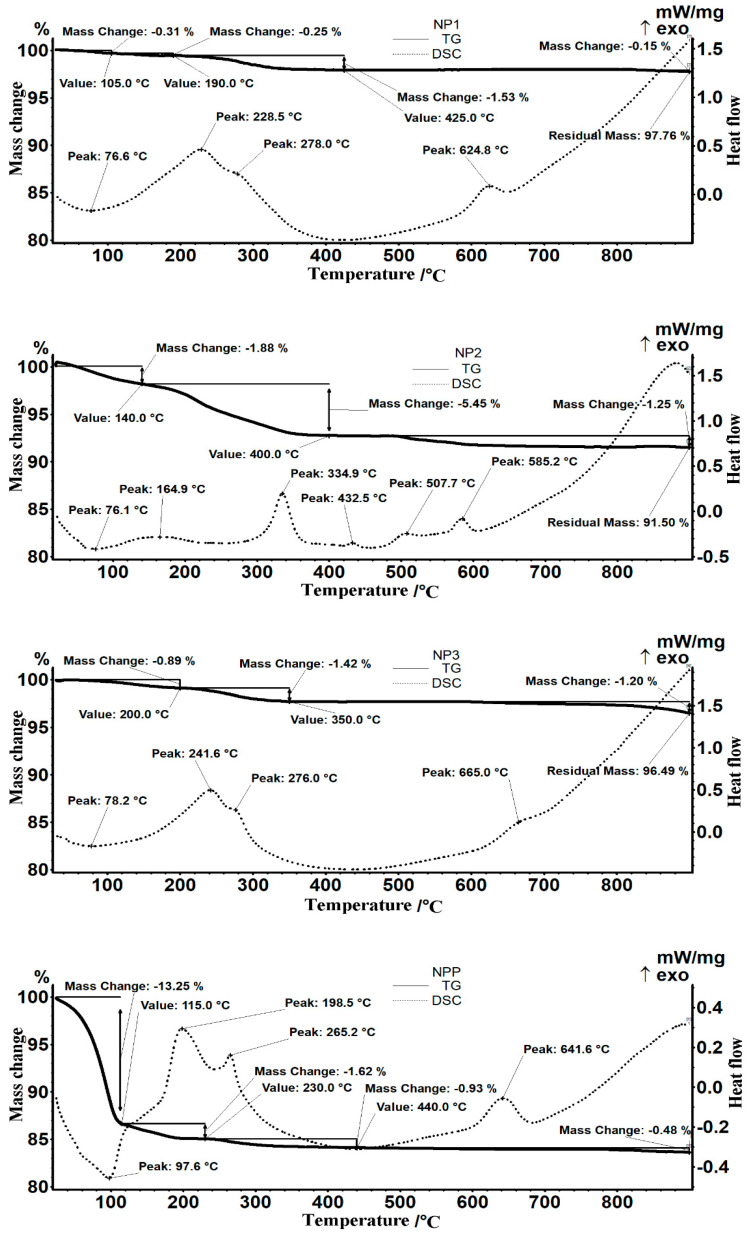
Detailed thermal diagrams for nanoparticles: NP_1_, NP_2_, NP_3,_ and a sample of nanoparticles after processing (NPP).

The thermal analysis generated the results materialized in the thermolysis curves from Figure 4 and Figure S5 (in Supplementary Materials), representing the initial nanoparticles’ thermal behavior. For comparison, it is also shown as an example the TG curve of the nanoparticles after their usage in the permeation process.

**Figure 5:**
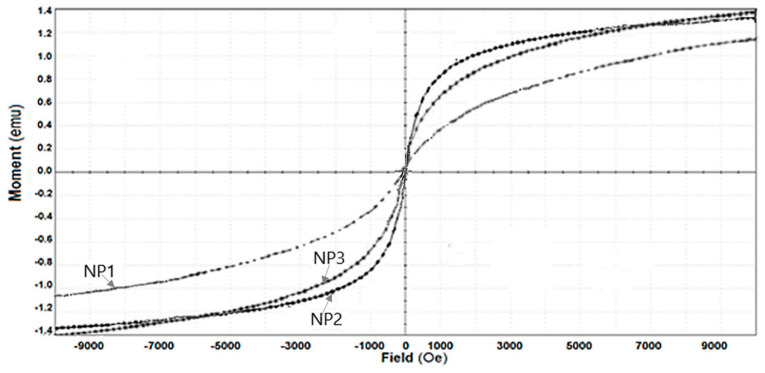
The magnetization diagrams for NP_1_, NP_2_, and NP_3_.

The magnetization diagrams for the prepared nanoparticles are shown in Figure 5.

**Figure 6:**
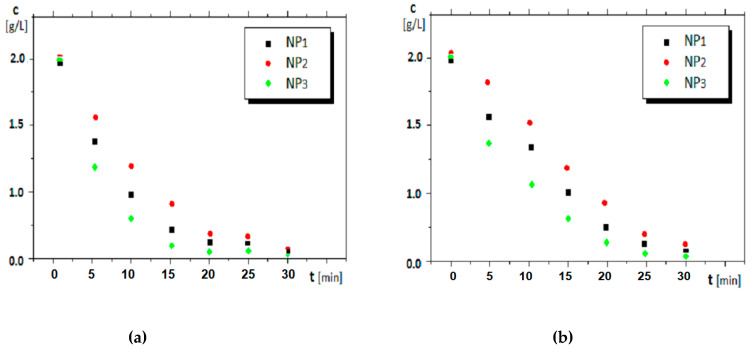
The source phase variation during the operation at a constant magnetic stirring regime (40 oscillations/min) for the three types of nanoparticles in *n-*decanol: (**a**) *o*-nitrophenol and (**b**) *m*-nitrophenol.

The magnetization curves for the prepared nanoparticles shown in Figure 6 were obtained at room temperature using a magnetometer that measures vibrations (SQUID Magnetometer, VSM). These diagrams show that the magnetite samples had a degree of magnetic saturation: 1.3 emu/g (NP1), 1.15 emu/g (NP2), and 1.40 emu/g (NP3). The magnetization curves indicate that the samples are superparamagnetic due to the absence of a hysteresis loop (Figure 6).

**Figure 7:**
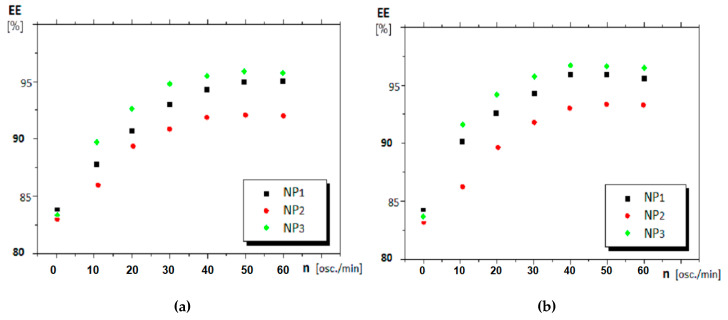
The variation of extraction efficiency for *o-*nitrophenol after 20 min of operating with nanoparticles dispersed against the stirring regime in the two considered alcohols: (**a**) *n*-octanol and (**b**) *n*-decanol.

**Figure 8:**
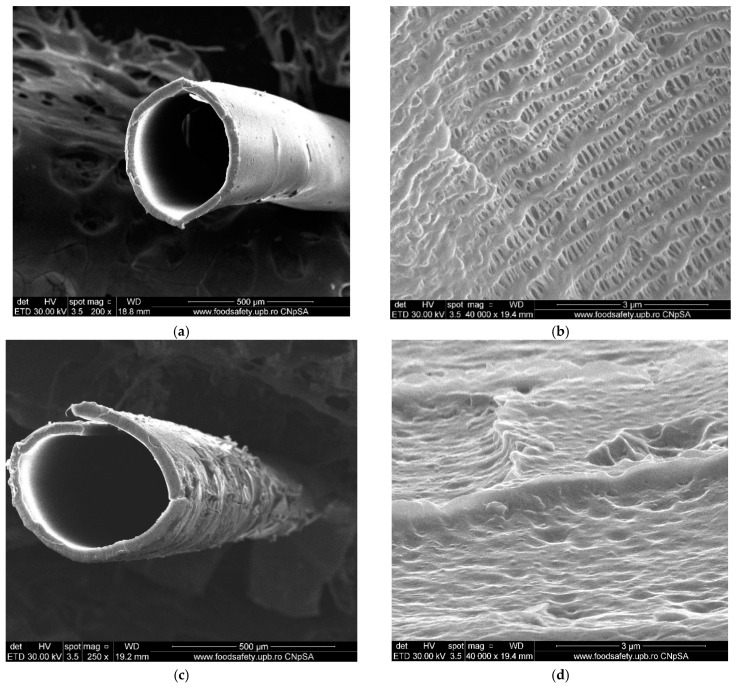
SEM images of the morphological aspect of the membrane support: (**a**) cross-section of the microporous polypropylene; (**b**) the surface of the microporous polypropylene; (**c**) a cross-section of the polypropylene fiber impregnated with nanoparticles dispersion; and (**d**) surface of the polypropylene impregnated with nanoparticles dispersion.

The results recorded for the nitrophenols pertraction are presented in Figure 7 and Figure 8.

The experimental data for the flux (J) and the extraction efficiency (EE) for *o-* and *m*- nitrophenols pertraction using the polypropylene supported liquid membranes based on *n*-alcohols (C_8_ and C_10_) and nanoparticles containing silver and iron oxide are presented in Table 2. The operating time was 30 min at 40 oscillations/min.

## 4. Discussion

Numerous studies showed over time that the membranes could be homogeneous or heterogeneous; with symmetric or asymmetric structures; solid, or liquid; neutral, or positively/negatively charged; as well as porous or dense [46,47]

The term “membrane” includes an impressive variety of materials and structures. From this large family of membranes, we chose the liquid membranes in *n*-octanol and *n*-decanol in which were dispersed nanoparticles containing silver and iron oxide, on a hollow-fiber microporous support—Figure 8.

The pores of hollow-fiber membranes (Figure 8b,d) are filled with the dispersion of nanoparticles in the chosen *n*-alcohol.

**Figure 9:**
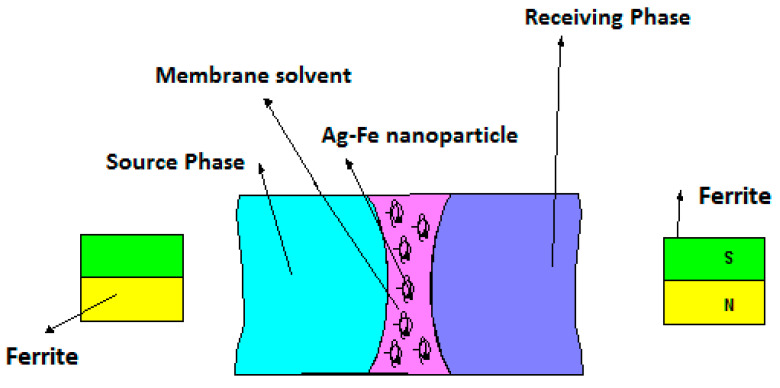
Schematic representation of the membrane system with magnetic iron oxide–silver nanoparticles onto the polypropylene fibers support.

The choice of membrane support and dispersion of magnetic nanoparticles containing iron oxide and silver in *n*-alcohols as a membrane medium (Figure 9) ensured a decrease of the membrane thickness that resulted in a reduced diffusion path of the permeate (nitrophenol), an increase of the mass transfer surface per volume unit, as well as a convective transport due to the magnetic stirring.

The choice of the sodium hydroxide solution as a reactant in the receiving phase took into account two factors, namely: the capacity of the reactant to convert the dissolved species that permeated in the receiving phase into impermeable forms, maintaining the concentration gradient [50]; and the insolubility of nitrophenol, as nitrophenolate, in the membrane phase [51].

### 4.1. Obtaining and Characterizing the Nanoparticles

The magnetic nanoparticles and magnetic nanocomposites led to a high interest in the supported liquid membranes technique [45]. The separation system used in this work was realized with the synthesized membrane material that contained iron oxide and silver nanoparticles with dimensions between 10 and 50 nm (Figure 3a–c).

The necessary silver for the nanoparticles’ preparation was obtained by the recuperative separation of the silver from the didactic and research activities developed in the University Politehnica of Bucharest (see Table S1). Annually, approximative 1400 g of waste containing silver chloride is obtained.

The raw materials containing silver, depending on the source and composition (Table 3), could be treated for solubilization with excess hydrochloric acid, ammonia, or sodium thiosulfate, according to the following reactions (I–III):

AgCl + HCl ⇆ H[AgCl_2_]  (I)

AgCl + 2NH_3_ ⇆ Ag (NH_3_)_2_ Cl  (II)

AgCl + 2Na_2_S_2_O_3_ ⇆ Na_3_Ag(S_2_O_3_)_2_ + NaCl  (III)

Considering the silver raw material composition and the collecting method applied (Table 3), only source 1 (AgCl from Cl^−^ titration) was used to study nanoparticle synthesis. Of course, all sources can be considered for silver recycling, but each requires a specific purification procedure, as suggested by the reactions (I-III).

In the obtained solutions there are present the complex ions: [AgCl_2_]^−^, [Ag(NH_3_)_2_]^+^, and [Ag(S_2_O_3_)_2_]^3−^, that are characterized through instability constants defined as:

(4) $$\beta_{1}=\frac{\left[MA\right]}{\left[M\right]\cdot \left[A\right]}\;$$

where *M* represents Ag, and *A* is the ligand.

The instability constants of the complexes are necessary for assessing the discharge potential of silver. For obtaining the electrolytic silver, cyclic voltammetry using pure iron electrodes and a platinum reference electrode at a potential swept between −0.5 and +1.23 V was applied.

The electrolysis experiments with soluble iron electrodes performed for all complexes: [AgCl_2_]^−^, [Ag(S_2_O_3_)_2_]^3−^, and [Ag(NH_3_)_2_]^+^ led to three types of nanoparticles.

The following reaction steps are known for the iron electrolysis mechanism in electrolyte without interfering electroactive species [21,22,23,24,25]:

Fe ⇆ Fe^2+^ + 2e^−^  (IV)

Fe^2+^ ⇆ Fe^3+^ + 1e^−^  (V)

H_2_O ⇆ 2H+ + 2e^−^ + 1/2 O_2_  (VI)

2H_2_O + 2e^−^ ⇆ H_2_ + 2HO^−^  (VII)

Fe^3+^ + 3HO^−^ ⇆ Fe(OH)_3_ (s)  (VIII)

3Fe(OH)_3_ (s) + H^+^ + e^−^ ⇆ Fe_3_O_4_(s) + 5H_2_O  (IX)

The silver discharge potential, which is affected by the equilibrium constants of the reactions (I–III), imposes the electrolysis potential to which the three silver complexes, used as electrolytes, could be discharged.

According to the XRD experimental data, the crystalline morphology of the nanoparticles was more specific to maghemite (Figure S4). This observation was sustained by the microscopy investigations when SEM images with specified dimensions of 10–50 nm (Figure 3a–c) were obtained. The nanoparticles contained mainly Fe and silver traces (see EDAX results on Figure S3a–c, correlated with Table 4).

As EDAX represents a localized analysis, a global analysis was performed for each of the three nanoparticle types, which were mineralized using specific reactions [64,65]. The investigation was done by UV-Vis spectrophotometry, and the data obtained were validated through atomic absorption analysis as presented in Table 5[66,67].

The data in Table 4 show that NP_3_ nanoparticles have the highest content in silver and NP_2_ has the lowest, while the atomic ratio Fe/O is 1:2.51 for NP_1_, 1:1.44 for NP_2,_ and 1:1.91 for NP_3_. Given that in pure oxides, the magnetite Fe/O ratio is 1.33 and that of maghemite 1.5, the deviation of the experimentally recorded values show clearly that there are adsorbed and chemo-absorbed hydroxyl residues from the environment on the obtained nanoparticle surface.

The global analysis of the nanoparticle samples highlighted the presence of silver. The overall concentration (Table 5) differed insignificantly compared to the values recorded on the surface of the nanoparticles (Table 4). These data showed that the nanometric samples have nanoparticles containing the two oxides, iron (majority) and silver oxides.

Detailed thermal analysis diagrams (Figure 4) for the nanoparticles: NP_1_, NP_2_, NP_3,_ and the sample of particles after processing (NPP) highlighted that the surfaces of the nanoparticles are covered with both complexing or adsorbed hydroxyl groups and traces of substances from synthesis (especially for NP_2_) or after pertraction procedure (NPP).

Thus, the following aspects were notable:

For the NP_1_ sample: In the interval 30–190 °C, the first mass losses occur (0.3%, then 0.25%), accompanied by an endothermic effect with a minimum at 76.6 °C. The mass loss is caused by water removal from the sample and possibly by removing some -OH groups on the nanoparticles’ surface.

In the range 190–425 °C, the recorded mass loss is 1.53%, accompanied by several weak, overlapping exothermic effects, with two maxima at 228.5 °C and 278 °C. In principle, the chemical residues from iron oxide synthesis can be degraded, or the precursors can be decomposed. However, the presence of silver nanoparticles could explain the exothermic peak from 278 °C, as within the 200–300 °C range, its crystallization may occur. The higher exothermic effect recorded at 228.5 °C can be attributed to the transformation of magnetite into maghemite (oxidation process). Considering the thermal effects occurring, all the reactions in the mentioned range are oxidative degradation processes.

An exothermic effect is observed on the DSC curve at 624.8 °C. This thermal effect represents the phase transformation of maghemite into hematite. It is a phase-specific transformation that is encountered in most samples containing magnetite. Still, this maximum position depends on many factors: nanoparticle size, shape, synthesis method, etc.

Then, between 425 and 900 °C, a new 0.16% mass loss occurs, which is insignificant in principle. The residual mass is 97.76%.

For the NP_2_ sample: In the first thermal decomposition stage within the temperature range RT–140 °C, the sample loses 1.88% of the initial mass. The process is accompanied by an endothermic effect with a minimum of 76.1 °C. Most likely, the sample loses traces of water.

In the range of 140–400 °C, the sample mass decreased by 5.45%. Several weak exothermic effects accompany the process, the most significant being at 334.9 °C, when possibly the degradative oxidation of chemical species traces remained absorbed on the particle surface may occur. It is also equally possible for -OH groups to condense and eventually to change the sample crystallinity.

After 400 °C, the sample loses 1.25% by the end of the analysis (900 °C), the process being characterized by the presence of several weak exothermic effects. The residual mass is 91.50%.

For the NP_3_ sample: In the interval 30–200 °C, the first mass loss occurs (0.89%), accompanied by an endothermic effect with a minimum at 78.2 °C. The loss of mass is due to the removal of water and possibly some -OH groups adsorbed on the nanoparticle surface.

In the range of 200–350 °C, the recorded mass loss is 1.42%, and there are present two overlapping weak exothermic effects, with the highest decomposition rates at 241.6 °C and 276 °C. Similar to the NP_2_ thermal analysis, traces of chemical species left from the magnetite synthesis can be degraded within this temperature interval. The maximum at 241.6 °C could be attributed to the transformation of magnetite into maghemite, while the exothermic process from 276 °C could be assigned to the silver nanoparticles crystallization.

An exothermic effect as a shoulder at 665 °C is observed on the DSC curve. It can be attributed to the maghemite–hematite phase transformation. Between 350 and 900 °C, there is a 1.2% mass loss, mainly after 750 °C, caused by the inorganic impurities that decompose at higher temperatures. The residual mass is 96.49%.

For the postprocessing sample (NPP): The sample loses 13.25% of its mass in the RT–115 °C range when an endothermic effect with a minimum at 97.6 °C is present. At this stage, traces of water or solvent weakly bound to the nanoparticles’ surface are removed. Since the sample loses mass almost as soon as heating begins, it is suggested that the adsorption of water molecules is favored by the adsorption of organic compounds from processing.

In the interval 115–230 °C, the sample loses 1.62% through a process accompanied by an exothermic effect with a maximum at 198.5 °C. Then, between 230 and 440 °C, the sample undergoes a second exothermic degradative oxidation process, losing 0.93% of its mass, with a maximum rate at 265.2 °C. Oxidation (burning) processes of substance traces absorbed on the surface could be assigned to the last two steps. Both the oxidation of magnetite and silver is done with a mass increase that could be masked by the mass loss recorded between 115 and 440 °C.

An exothermic effect is observed on the DSC curve at 641.6 °C, representing the maghemite phase transformation into hematite. The residual mass is 83.63%.

The after-processing nanoparticle sample was also characterized morphologically, and the dimensions were evaluated by scanning electron microscopy (Figure S6 from Supplementary file). There is a marked aggregation of nanoparticles after processing, which is justified by the higher amount of adsorbed water and organic compounds (nitrophenol and/or *n*-alcohol), as previously evidenced through thermal analysis (Figure 4-NPP).

### 4.2. Process Performance of the Membrane System with Silver–Iron Oxide Magnetic Nanoparticles in n-Alcohols on Polypropylene Fiber Support

**Figure 10:**
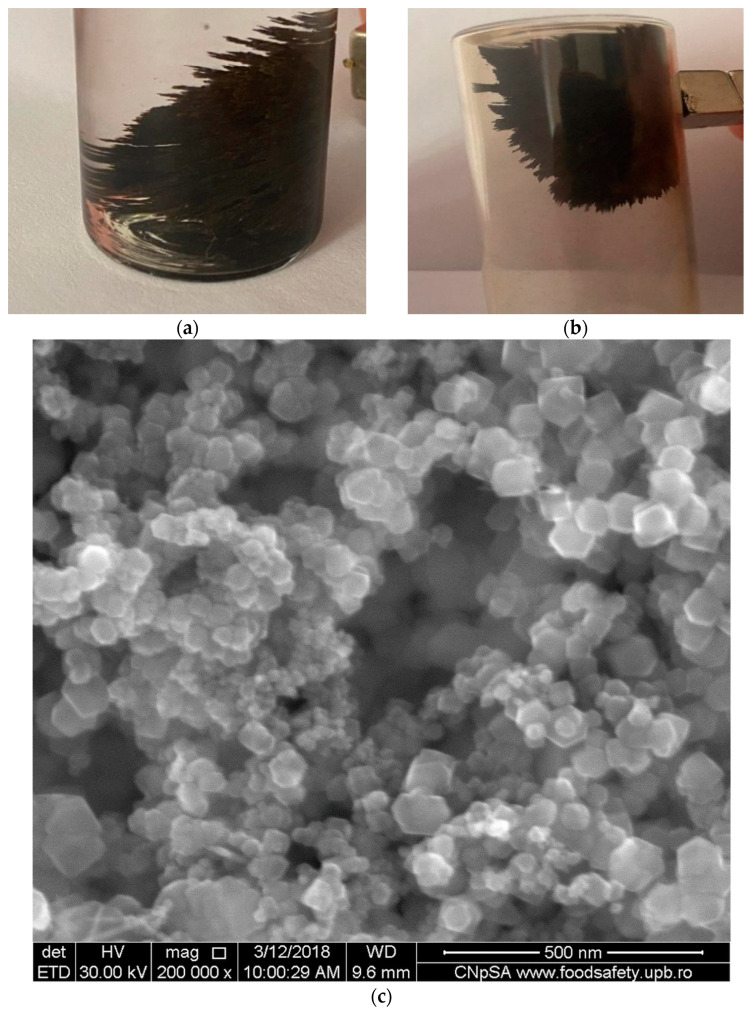
The unitary behavior of the silver–iron oxide membrane material: (**a**) at separation from aqueous solution; (**b**) in the dry state (in the air); (**c**) SEM detail presentation.

The magnetic membrane material based on silver–iron oxide used for the nitrophenols transportation presented a unitary behavior when interacting with the magnetic field (Figure 10). Such behavior was observed both when separated from the aqueous solution (from its preparation solution) (Figure 10a) and in the dry state (Figure 10b).

The superparamagnetic behavior of silver and iron oxide nanoparticles is remarkable, interacting strongly even when a small magnetic element is used (Figure 10a,b). These properties are due to the narrow distribution of nanoparticle sizes (Figure 3a–c). The details show that they are aggregates of nanoparticles with dimensions of several tens of nanometers (Figure 10c).

The choice of nitrophenols as a target chemical species for evaluating performances of the prepared membrane by incorporating the nanoparticles dispersions onto polypropylene microporous support is argued by the importance of phenols in recovery studies from various aqueous solutions; the knowledge of operating parameters (initial phase solution concentration, pH of aqueous phases, membrane solvents characteristics); and accessibility of analysis methods.

The pertraction mechanism of nitrophenols separation through supported liquid membranes is well-known [56,57,58,59]. It implies the existence of a pH gradient between the source and the receiving phase (see Figure S7). In our case, the liquid membrane is the silver–iron oxide nanoparticles dispersion that is stirred by an oscillating magnetic field to generate a convective transport.

During the experiments, the pH was 1 for the source phase and 13 for the receiving phase. As already mentioned, the membrane was a dispersion of silver–iron oxide magnetic nanoparticles in *n*-medium alcohols supported on the microporous polypropylene hollow fibers. The oscillating magnetic field was created by sliding a ring-like ferrite along the pertraction module (Figure 2). In Table 2, Figure 6 and Figure 7 are presented the results obtained in the nitrophenols extraction under the chosen operating conditions.

#### 4.2.1. The Effect of the Nanoparticle Composition

Under the same conditions of magnetic stirring, 40 oscillations per minute, the decrease of the nitrophenols concentration in the source phase is accentuated (Figure 6), and more obvious for *o*-nitrophenol (Figure 6a) than for *m-*nitrophenol (Figure 6b), achieving after 20–25 min of operation a separation with an efficiency over 90%. The decrease in nitrophenol concentration is determined by the Ag nanoparticles concentration (Table 2). Thus, the extracted nitrophenol concentration is higher for nanoparticles with higher Ag content (Table 2 and Table 5, and Figure 6). This variation overlaps with that of the dispersion’s saturation magnetization, as shown in Figure 5.

**Figure 11:**
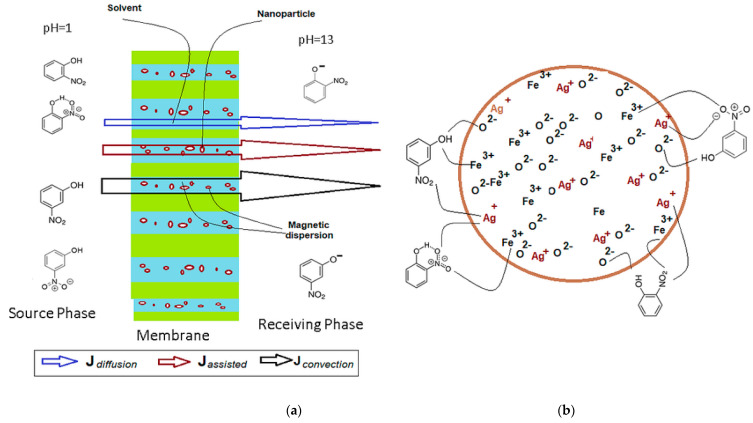
The pertraction mechanism through supported liquid membranes. (**a**) membrane system and specific fluxes (J); (**b**) assisted nanoparticle transport mechanism for *m*- and *o*-nitrophenol.

The results obtained indicate the participation of oxide nanoparticles in convective transport and interaction with the separated nitrophenol (Figure 9 and Figure 11a,b).

The interaction of the metal ions from the nanoparticles with the nitrophenol is the only explanation for the superior results obtained both when the system is under the action of an oscillating magnetic field and without being magnetically stirred (Figure 8a,b—at zero oscillations).

#### 4.2.2. The Effect of the Oscillating Magnetic Field

The magnetic oxide material unitary characteristics are also transferred to the impregnated fibers (Figures S1 and S2c,d), thus interacting with the magnetic field outside the hollow-fiber membranes.

In the oscillating magnetic field, the membranes move similarly to the jellyfish terminations (Figure S2c), generating convection between the fibers and the source phase. Consequently, the magnetic nanoparticles oscillate, generating convection in the membrane solvent inside the fiber wall.

Thus, the nitrophenol extraction was performed in an oscillating magnetic field (Figure 7). During the separation procedure, it was highlighted the importance of saturation magnetization. In recent work, L. Upadhyaya et al. demonstrated the influence of the magnetic field on the permeate flux profiles in some polymeric membranes with iron-oxide core [68]. The oscillating magnetic field causes a significant increase in the extraction efficiency of *o-*nitrophenols from the source phase, more accentuated within the interval of 30–40 oscillations per minute. After 40 oscillations/min, the extraction efficiency recorded a plateau, which indicates that the nanoparticles can no longer accompany the ferrite in its movement. This observation is substantiated by nanoparticle dispersion in *n-*octanol (with lower saturation magnetization) with noticeably different behavior from nanoparticle dispersion in *n-*decanol. It is a competition between the saturation magnetization of the nanoparticle dispersions and the viscosity of the membrane solvent. Such behavior highlights that the nanoparticles with higher magnetization give better extraction performance in terms of flux and extraction efficiency, especially in *n-*decanol (Table 2).

Overall, the extraction performance, expressed as the flux and extraction efficiency, increases in order:

EE_NP3_ > EE_NP1_> EE_NP2_

J_NP3_ > J_NP1_> J_NP2_

for the flux and extraction efficiency, for both dispersions, *n-*octanol, and *n-*decanol (Figure 6 and Figure 7, and Table 2).

The nanoparticles with a higher silver content determine better performance leading to an increased extraction efficiency due to the convective effect caused by the oscillating magnetic field and the silver-assisted nitrophenols transport.

Thus, it is possible to suggest the dependence of the total flow of nitrophenol through the membrane on diffusion–solubilization, convection, and silver assisted transport, as follows:

(5) $$J_{\mathrm{total}}=J_{\mathrm{diffusion}}+J_{\mathrm{convection}}+J_{\mathrm{assisted}}$$

In other words, the total flux cumulates the effect of the solvent, the magnetization, and the silver content, according to:

(6) $$J_{\mathrm{nitrophenol}}=J_{\mathrm{solvent}}+J_{\mathrm{NP}}+J_{\mathrm{Ag}}$$

Although evaluating the convection contribution on the total nitrophenol flux can be appreciated by the difference between the extraction efficiency at the initial moment, when the system is not magnetically agitated (*n*_osc_ = 0) and, respectively, at a vigorous stirring (*n*_osc_ ≠ 0) (Figure 7a,b), the contribution of adsorption assistance on nanoparticles (Figure 11 b) is more difficult to be assessed. However, the interaction of the nanoparticles, through their ions (Ag^+^, Fe^3+^ and O^2−^), with the considered nitrophenols is highlighted by the significant differences in extraction and flow efficiency for *o*-nitrophenol and *m*-nitrophenol, respectively (Figure 6 and Figure 7, Table 2). The multiple interactions of *m*-nitrophenol with nanoparticles (Figure 11b) causes an accumulation inside the membrane and a slower release in the receptor phase that lead to smaller fluxes than *o*-nitrophenol, which through the internal interaction (hydrogen bonding), allows weaker adsorption on the nanoparticles surface, and a faster release in the receiving phase (Table 2). The observations are also justified by the surface composition (Figure S3a–c, Table 3) and the different magnetizations (Figure 5).

## 5. Conclusions

In the present paper, the transport *o-* and *m-*nitrophenols was performed using *n-*octanol/*n*-decanol dispersion of magnetic iron oxide and electrolytic recovered silver nanoparticles supported on microporous polypropylene hollow fibers.

The procedure for recovering the necessary silver involved the solubilization of the available silver chloride in hydrochloric acid, sodium thiosulfate, and ammonia. Then, the obtained electrolytes: [AgCl_2_]^−^, [Ag(S_2_O_3_)_2_]^3−^, or [Ag(NH_3_)_2_]^+^ were subjected to cyclic voltammetry (potential scanning from −0.5 to +1.23 V), using a cell with pure iron anode and cathode and a platinum reference electrode. Starting from each silver electrolyte complex, three types of nanoparticles were obtained, namely NP_1_, NP_2_, and NP_3_, whose dispersion in *n-*octanol or *n-*decanol provided the membrane material for the liquid membranes supported on microporous polypropylene hollow fibers.

Separation of the target substances (*o-*nitrophenol and *m-*nitrophenol) in an oscillating magnetic field showed that the overall extraction performances (extraction efficiency (EE) and flux (J)) increased in the order: EE_NP3_ > EE_NP1_ > EE_NP2_, respectively: J_NP3_ > J_NP1_ > J_NP2_. The efficiency of separation using silver–iron oxide magnetic nanoparticles is correlated with their saturation magnetization and silver content. Thus, it is possible to suggest a dependence of nitrophenol total flow through the membrane on diffusion, convection, and silver-assisted transport. Or, otherwise expressed, the total flux cumulates the effect of solvent magnetization and silver content.

## Figures and Tables

**Table 1:** Main features of the polypropylene fibers (GOST Ltd.).

Material	Polypropylene (PP)
Porosity	40–50%
Dimension of pore	0.002–0.2 µm
External diameter	0.45 mm
pH	1–14
T (°C)	T_max_ = 50 °C
Fascicle dimensions	Φ 25 × 750 mm^2^
Filtration surface (fascicle)	1.0 m²
Operation pressure	0.1–0.4 bar
The average flow of permeate	10–15 L/m²h

**Table 2:** The flux (J) and the extraction efficiency (EE) for *o-* and *m*-nitrophenols pertraction.

Liquid Membrane with Nanoparticles		NP_1_		NP_2_		NP_3_	
		C_10_–OH	C_8_–OH	C_10_–OH	C_8_–OH	C_10_–OH	C_8_–OH
**J**, **(g h^−1^ m^−2^)**	*o*-Nitrophenol	194 + 2	186 + 2	187 + 2	172 + 2	198 + 2	192 + 2
	*m*-Nitrophenol	188 + 2	182 + 2	180 + 2	168 + 2	191 + 2	186 + 2
**EE** (%)	*o*-Nitrophenol	97 + 1	93 + 1	93.5 + 1	86 + 1	99 + 1	96 + 1
	*m*-Nitrophenol	94 + 1	91 + 1	99 + 1	84 + 1	95.5 + 1	93 + 1

**Table 3:** Composition of silver raw materials depending on their origin.

No.	Silver Source	Composition (%)					
		Ag	Cl	Br	I	S	Other
1.	Titration of chloride ions	75.26	24.73	-	-	-	-
2.	Titration of chloride and iodide ions (separately and in a mixture)	74.38	20.10	-	5.51	-	-
3.	Photo labs (classical processed)	67.43	10.32	12.67	-	9.17	Organic impurities
4.	Determination of drinking water characteristics; general cations separation; research activities.	65.88	20.03	9.24	4.54	-	Inorganic impurities (metallic ions)

**Table 4:** EDAX results for the prepared nanoparticles.

The Specific Spectral Line Used	Nanoparticles					
	NP_1_		NP_2_		NP_3_	
	Weight %	Atomic %	Weight %	Atomic %	Weight %	Atomic %
**O** K	41.52	71.41	29.2	59.05	38.02	65.39
**Ag** L	0.93	0.24	0.21	0.06	1.12	0.28
**Fe** K	57.55	28.35	70.59	40.89	60.86	34.33

**Table 5:** UV-Vis and atomic absorption spectrophotometric analysis of nanoparticles.

The Specific Metal	Nanoparticles					
	NP_1_		NP_2_		NP_3_	
	UV-Vis	AAS	UV-Vis	AAS	UV-Vis	AAS
**Ag** (%)	1.04 + 0.04	1.038 + 0.003	0.23 + 0.07	0.228 + 0.005	1.20 + 0.03	1.219 + 0.001
**Fe** (%)	56.88 + 0.07	56.854 + 0.005	69.98 + 0.08	70.146 + 0.009	59.87 + 0.04	59.900 + 0.007

## Data Availability

Data is contained within the article or Supplementary Materials.

## Supplementary Materials

The following are available online at https://www.mdpi.com/article/10.3390/nano11051204/s1, Figure S1: Preparation of the impregnated hollow fiber with nanodispersion; Figure S2: Details of the essential elements of the pertraction installation; Figure S3: EDAX diagrams; Figure S4: XRD diagram for the obtained nanoparticles; Figure S5: General thermal diagrams; Figure S6. SEM images of NPP nanoparticles; Figure S7: The pertraction mechanism through supported liquid membranes; Table S1: Silver sources (AgCl).
