# Supplementary material for: Accessible Silver-Iron Oxide Nanoparticles as a Nanomaterial for Supported Liquid Membranes
Source: Nanomaterials (Basel). 2021 May 1;11(5):1204. doi: 10.3390/nano11051204 (PMC8147404; doi:10.3390/nano11051204)
Supplement: Supplementary file 1 [file nanomaterials-11-01204-s001.zip › nanomaterials-1192425-SI-final.pdf]

Supplementary Materials

Accessible Silver-Iron Oxide Nanoparticles as a Nanomaterial for Supported Liquid Membranes

Ioana Alina Dimulescu (Nica) [1], Aurelia Cristina Nechifor [1,*], Cristina Bărdacă (Urducea) [1], Ovidiu Oprea [2], Dumitru Paşcu [1], Eugenia Eftimie Totu [1,*], Paul Constantin Albu [3], Gheorghe Nechifor [1] and Simona Gabriela Bungău [4]

1 Analytical Chemistry and Environmental Engineering Department, University Politehnica of Bucharest, 1-7 Polizu Street, 011061 Bucharest, Romania; oanaalinadimulescu@yahoo.com (I.A.D.); cristinabardaca@yahoo.com (C.B.); dd.pascu@yahoo.com (D.P.); gheorghe.nechifor@upb.ro (G.N.)
2 Department of Inorganic Chemistry, Physical Chemistry and Electrochemistry, University Politehnica of Bucharest, 1-7 Polizu Street, 011061 Bucharest, Romania; ovidiu73@yahoo.com
3 Horia Hulubei National Institute for R&D in Physics and Nuclear Engineering (IFIN-HH), Radioisotopes & Radiation Metrology Department (DRMR), 30 Reactorului Street, 023465 Magurele, Romania; paulalbu@gmail.com
4 Faculty of Medicine and Pharmacy, University of Oradea, Universităţii Street No.1, Oradea, County Bihor 410087, Romania; simonabungau@gmail.com
* Correspondence: aurelia.nechifor@upb.ro (A.C.N.); eugenia.totu@upb.ro (E.E.T.)

Preparation of Impregnated Hollow Fiber with Nanodispersion

In Figure S1 there are presented the main stages of preparing the impregnated hollow fibers. The bundle of microporous polypropylene fibers was immersed in the solution without inserting the sealing ends (made of polyvinyl chloride). After 24 h soaking, the bundle of polypropylene fibers impregnated with nanoparticles containing iron oxide and silver in the considered *n*-alcohol was recovered and washed for 10 min with deionized water.

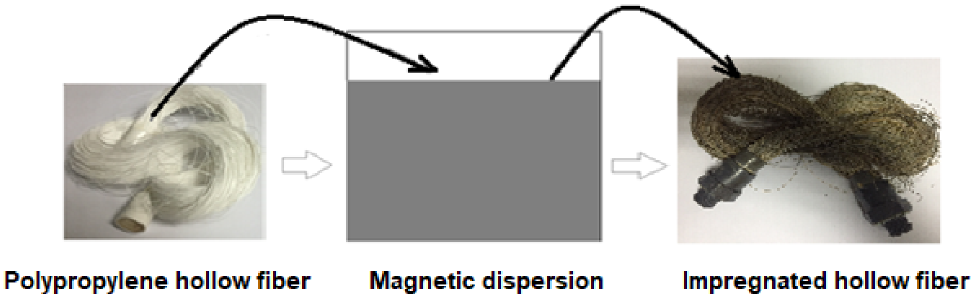

Figure S1. Preparation of the impregnated hollow fiber with nanodispersion.

Pertraction Module and its Principal Component

The central element of the pertraction installation is the fiber bundle (Figure S2a). When the fibers are impregnated, they are attracted by the magnetic field created by the working ferrite (Figure S2b) or other magnetic material (Figure S2c). The geometric characteristics of the working ferrite are given by the manufacturing company (Figure S2d).

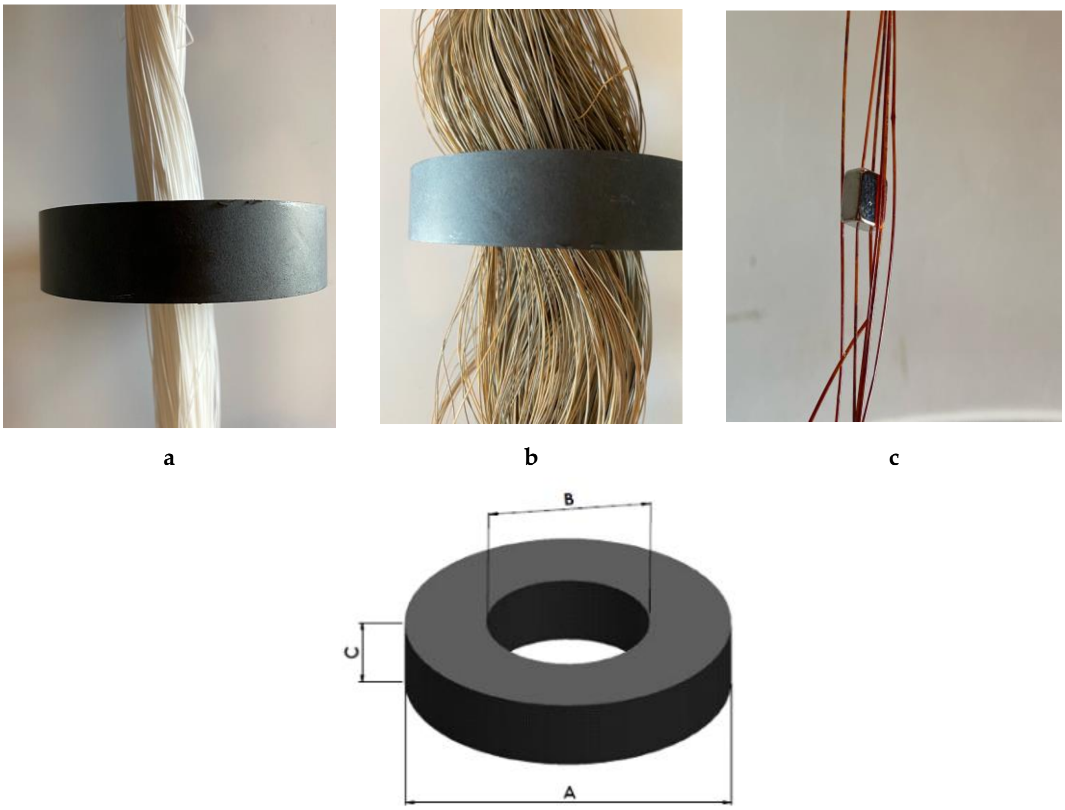

d

.

Figure S2. Details of the essential elements of the pertraction installation (**a**) non-magnetic hollow fiber bundle; (**b**) magnetic hollow fiber bundle; (**c**) magnetic hollow fiber proprieties; (**d**) annular ferrite: A=100mm; B=60mm; C=20mm.

Structural analysis

Figure S3 presents the EDAX analysis for the composite nanoparticles obtained using the electrolytic systems based on: $[AgCl_2]^-$ shorthand notation NP_1; $[Ag(S_2O_3)_2]^{3-}$ shorthand notation NP_2; $[Ag\ (NH_3)_2]^+$ shorthand notation NP_3.

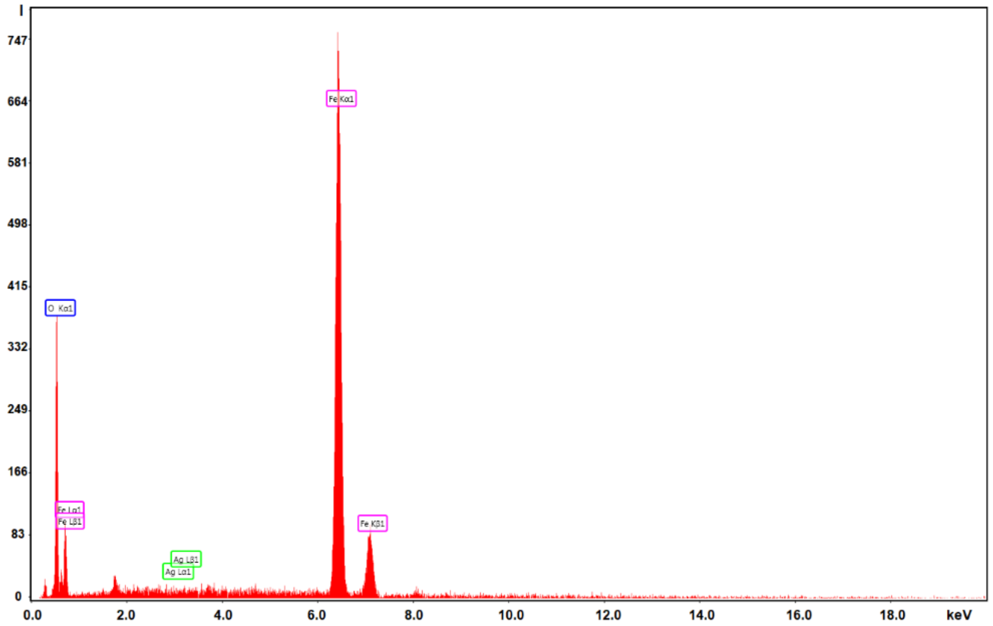
I
747
664
581
498
415
332
249
166
83
0
O Kα1
Fe Kα1
Fe Lα1
Fe Lβ1
Ag Lβ1
Ag Lα1
Fe Kβ1
0.0
2.0
4.0
6.0
8.0
10.0
12.0
14.0
16.0
18.0
keV

a

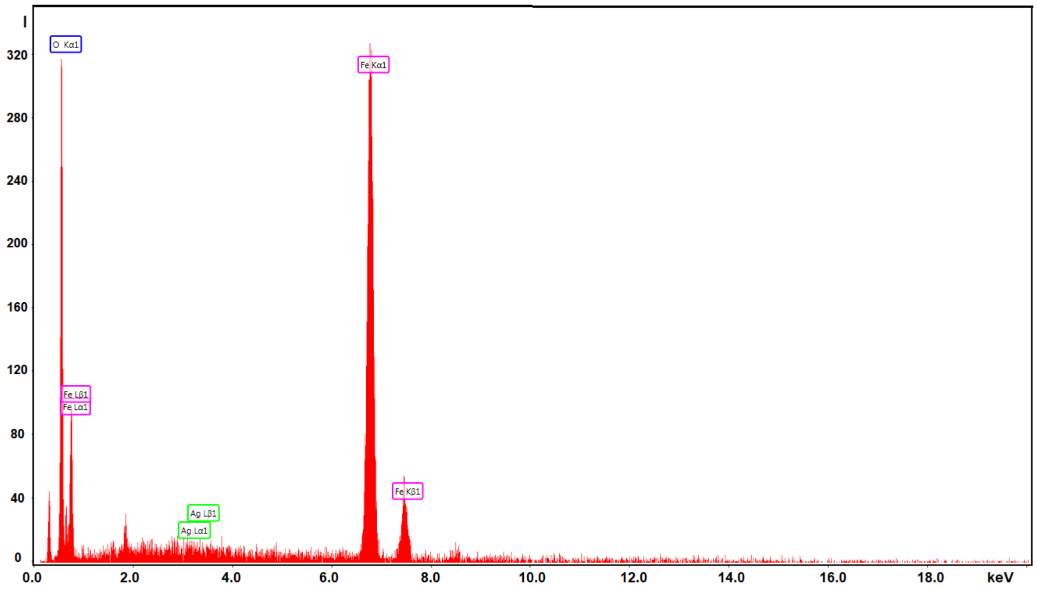
I
320
280
240
200
160
120
80
40
0
O Kα1
Fe Kα1
Fe Lβ1
Fe Lα1
Ag Lβ1
Ag Lα1
Fe Kβ1
0.0
2.0
4.0
6.0
8.0
10.0
12.0
14.0
16.0
18.0
keV

b

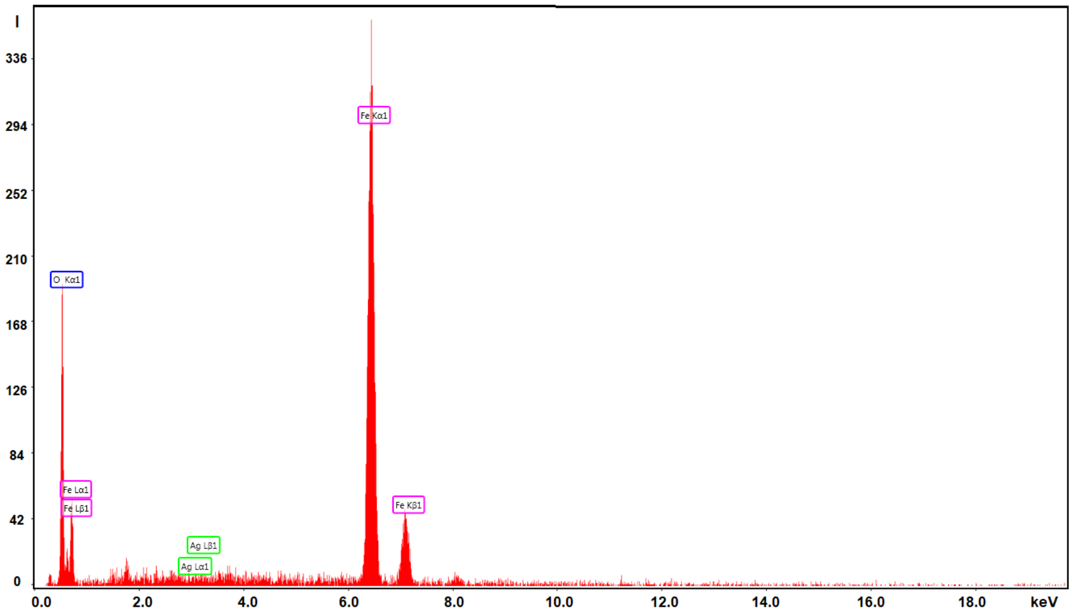

c

Figure S3. EDAX diagrams. (**a**). NP1 -EDAX; (**b**). NP2-EDAX; (**c**). NP3-EDAX.

XRD patterns for the obtained nanoparticles are presented in Figure S4. Comparing the experimental data to reference fingerprints, it was determined which phases are present in the analyzed samples.

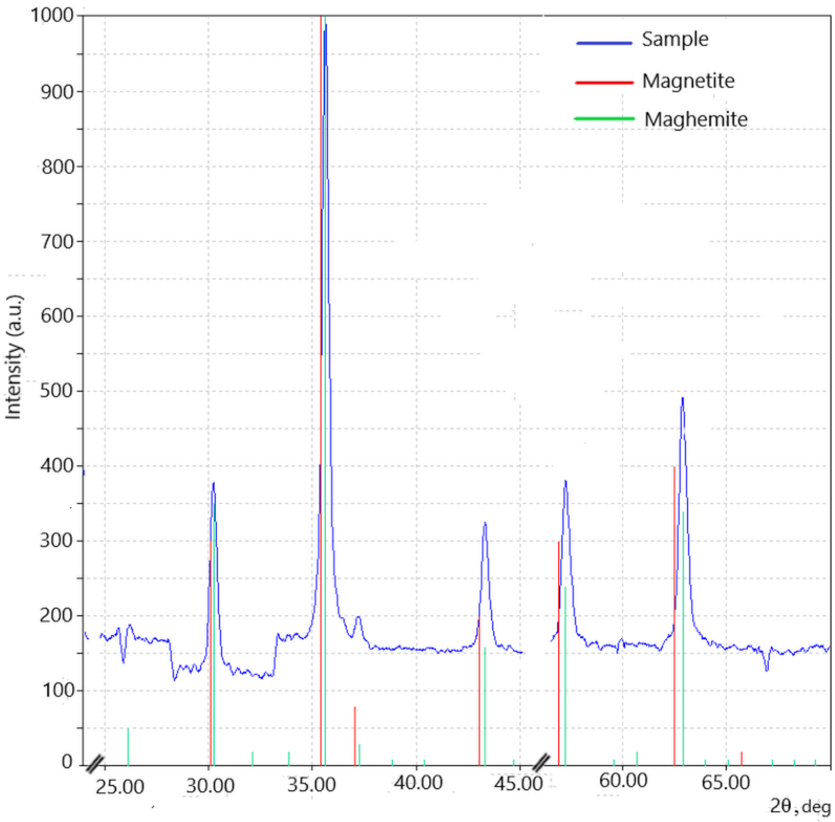

Figure S4. XRD diagram for the obtained nanoparticles.

Thermal analysis

Figure S5 presents the general thermogravimetric plots and DSC curves for the initial nanoparticles' and the nanoparticles after their usage in the permeation process (NPP).

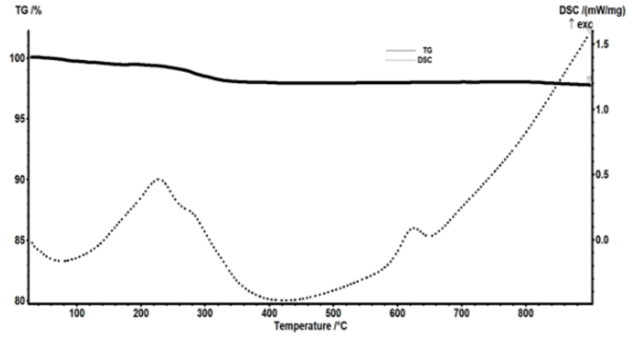

NP1

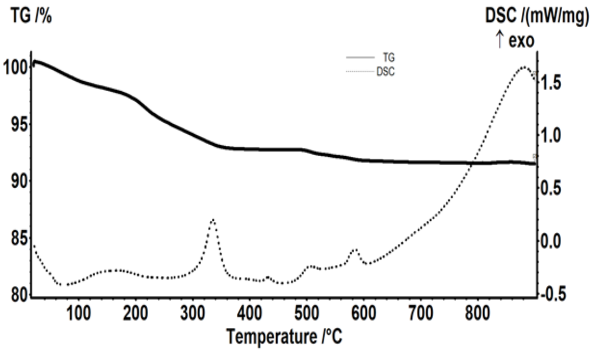

NP2

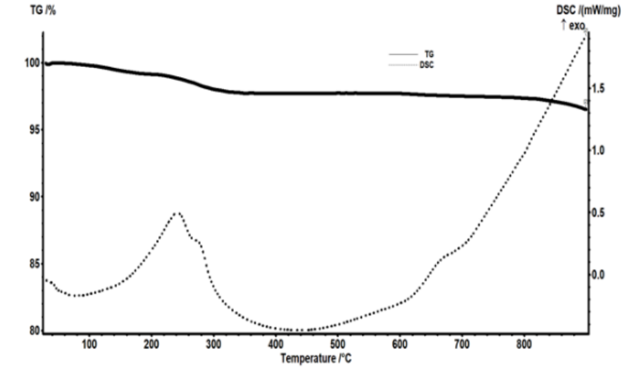

NP3

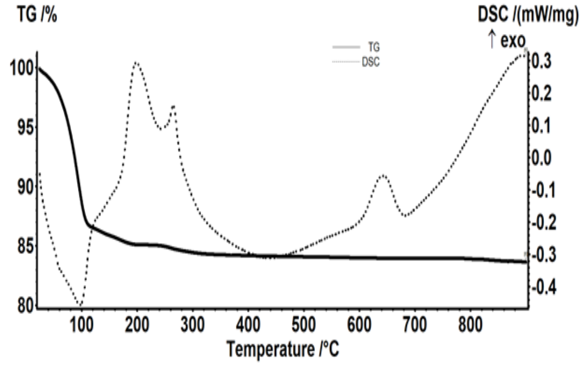

NPP

Figure S5. Thermal diagrams for the three nanoparticle types: NP1, NP2, NP3, and nanoparticles after permeation process, NPP.

SEM Investigation on the After-Processing Nanoparticle Sample

Figure S6 presents the morphological aspects of the after-processing nanoparticle sample. The SEM analysis allowed the determination of the nanoparticle dimensions, Figure S6b. There is a marked aggregation of nanoparticles after processing, which is justified by the higher amount of adsorbed water, as well as organic compounds (nitrophenol and/or n-alcohol), as previously evidenced through thermal analysis (Figure S5 -NPP).

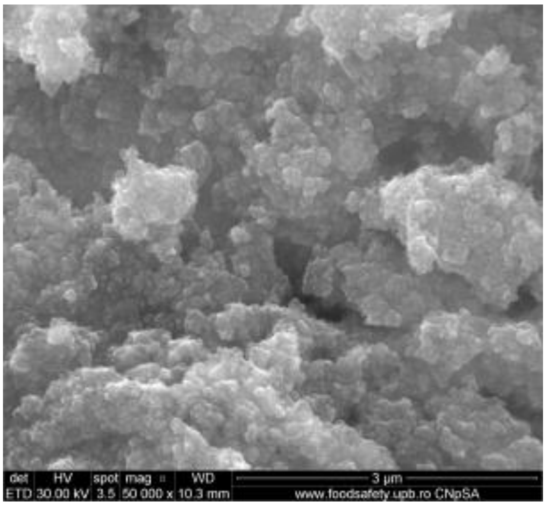

a

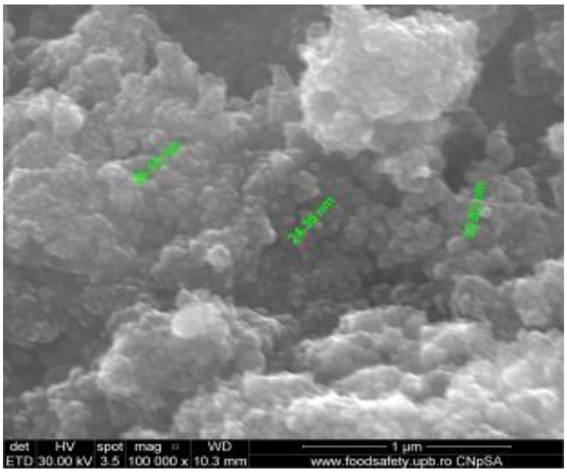

b

Figure S6. SEM images of NPP nanoparticles. (**a**) – morphology, (**b**) – characteristic dimensions.

Pertraction mechanism

The pertraction mechanism of nitrophenols separation through supported liquid membranes implies a pH gradient between the source and the receiving phase, as shown in Figure S7. In our case, the liquid membrane is the silver-iron oxide nanoparticles dispersion that is stirred by an oscillating magnetic field to generate a convective transport.

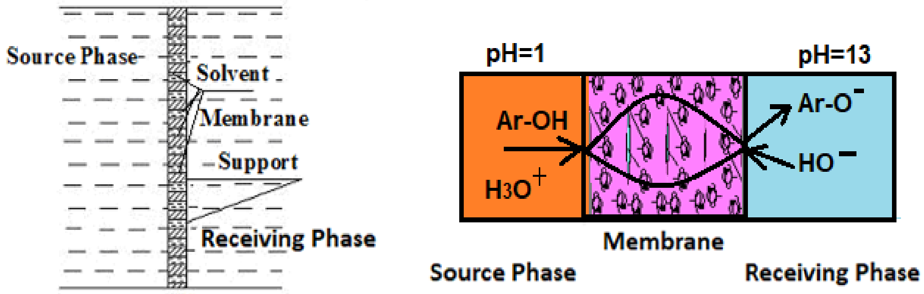

a b

Figure S7. The pertraction mechanism through supported liquid membranes. (**a**) membrane system; (**b**) transport mechanism.

Sources for the Recovered Silver

The necessary silver for the nanoparticles' preparation was obtained by the recuperative separation of the silver from the didactic and research activities developed in the University Politehnica of Bucharest. In Table S1 are presented the silver sources (AgCl) and the annual quantities generated.

Table S1. Silver sources (AgCl) and the annual quantities generated in University Politehnica of Bucharest

AgCl generating activity	Students number (Licence and Master studies)	AgCl quantity (g-dry substance)
**Volumetric titration of the chloride ions**	320	160
**Potentiometric precipitation titration of the chloride ions**	300	80
**Determination of the transport number of silver**	200	50
**Systematic cations separation for the V-th analytical group**	320	75
**General cations separation**	320	40
**Catalysts recovery**	50	180
**Membrane transport and separation**	40	25
**Determination of drinking water characteristics**	80	50
**Research activities**	500	300
**Photo labs**	35	400
**Total**		1370
